# Easily misdiagnosed delayed metastatic intraspinal extradural melanoma of the lumbar spine: A case report and review of the literature

**DOI:** 10.3892/ol.2013.1299

**Published:** 2013-04-10

**Authors:** LIN SUN, YUEMING SONG, QUAN GONG

**Affiliations:** Department of Orthopedics, West China Hospital, Sichuan University, Chengdu, Sichuan 610041, P.R. China

**Keywords:** metastatic melanoma, lumbar spine, extradural, delayed metastasis, lumbar stenosis, misdiagnosed

## Abstract

Metastatic melanoma of the spine usually occurs as vertebral metastatic melanoma or intramedullary spinal cord metastatic melanoma. The present study reports a case of easily misdiagnosed delayed metastatic intraspinal extradural melanoma of the lumbar spine. A 67-year-old female patient presented with lower back pain accompanied by progressive intermittent claudication. Magnetic resonance imaging (MRI) suggested compression of the lumbar spinal cord caused by an extradural mass. The mass showed T2-hypointensity, T1-hypointensity and slight enhancement following a gadolinium-contrast injection. The patient had been diagnosed with a vulvar melanoma 13 years previously and had also undergone a resection of this tumor. A current diagnosis of a lumbar stenosis resulting from hypertrophy of the ligamentum flavum was suspected. However during corrective surgery, a dark gray solid mass was observed. An L3 laminectomy and removal of the tumor was performed. The tumor was confirmed to be a malignant melanoma by histopathological investigation. The patient was treated with radiotherapy and immunotherapy. At the final 13-month follow-up, the patient showed no signs of recurrence. It may be concluded that an early diagnosis of metastatic melanoma was prevented by delayed metastasis, the location of the mass and its unusual appearance in MRI scans. In such cases, early surgical removal and an appropriate comprehensive treatment are critical for patient survival. These observations suggest that caution should be used in the diagnosis of similar cases.

## Introduction

Melanomas are malignant tumors prone to early metastasis, which occurs with a high incidence in the Caucasian populations (1). Almost any organ and structure may be involved in metastatic melanoma due to its ubiquitous spreading pattern. The most common forms of metastatic melanoma of the spine are vertebral metastatic melanoma and intramedullary spinal cord metastatic melanoma (2,3). Spine metastasis of melanoma carries the potential for difficult problems, such as pain, weakness and sensory deficit. However, there is few stuides describing intraspinal extradural metastatic melanoma. The present study reports a case of a delayed metastatic intraspinal extradural melanoma of the lumbar spine in a non-Caucasian patient. The diagnosis of the tumor was complicated by its delayed metastasis, location and unusual appearance in magnetic resonance imaging (MRI) scans. Written informed consent was obtained from the patient.

## Case report

A 67-year-old female patient was admitted with a six-month history of lower back pain accompanied by a two-month history of intermittent claudication. The patient had visited a community hospital when the lower back pain developed and was diagnosed with osteoporosis following assessment by radiography of the lumbar spine. Despite treatment with medication and physiotherapy, the patient’s symptoms did not subside. Two months prior to this admission the patient began to experience progressive intermittent claudication. The patient was referred to the West China Hospital (Sichuan University, Chengdu, China) as an MRI of the lumbar spine had been recommended three weeks prior to the admission. A neurological examination showed no notable changes in the function of the spinal nerve.

The patient had previously been diagnosed with a vulvar melanoma (13 years prior to the current admittance) and had undergone a melanoma resection at the West China Hospital. The patient received post-surgical radiotherapy. No local melanoma recurrence or other metastatic melanoma was reported prior to the present admission. The patient had been treated with anti-hypertensive drugs for 11 years, but had no other medical history.

**Figure 1:**
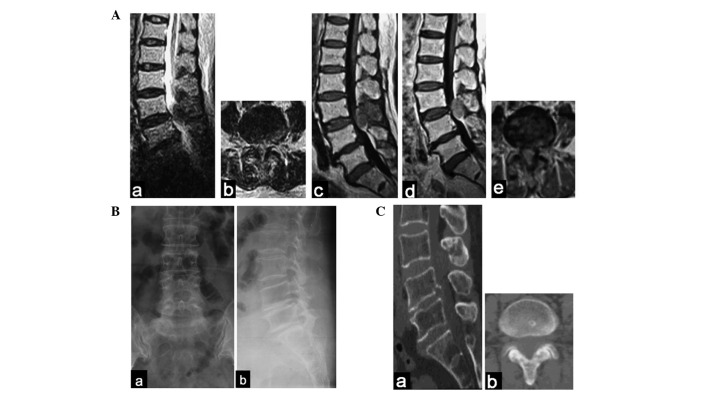
(A) At the L3–L4 level, MRI revealed an extradural posterior oval mass with (a and b) low signal intensity on T2-weighted images, (c) mixed low intensity on T1-weighted images and (d and e) slight enhancement images. (Ba and b) Radiography of the lumbar spine revealed no evident changes. (Ca and b) CT scans faintly revealed the mass. MRI, magnetic resonance imaging. Aa, T2/sagittal plane; Ab, T2/transverse plane; Ac, T1/sagittal plane; Ad, enhanced/sagittal plane; Ae, enhanced/transverse plane; Ba, posteroanterior; Bb, lateral; Ca, sagittal plane; Cb, transverse plane.

The current MRI revealed compression of the lumbar spinal cord in the spinal canal from the posterior direction caused by an extradural mass at the L3 and L4 level. The mass showed a low signal intensity on the T2-weighted images, a mixed low signal intensity on the T1-weighted images and slight enhancement following a gadolinium-contrast injection (Fig. 1A). Over the subsequent week, radiography and computed tomography (CT) of the lumbar spine were recommended. There were no clear changes in the radiograph (Fig. 1B). CT scans faintly revealed the mass (Fig. 1C).

Although the patient had a medical history of vulvar melanoma, a lumbar stenosis resulting from hypertrophy of the ligamentum flavum was suspected instead of metastatic melanoma due to the extended time-period (13 years) since the original vulvar melanoma, the location of the mass and its appearance in MRI scans. Treatment with medication was prescribed to the patient. However, two weeks later, the intermittent claudication was significantly aggravated so the patient was re-admitted. The laboratory data showed normal values.

**Figure 2:**
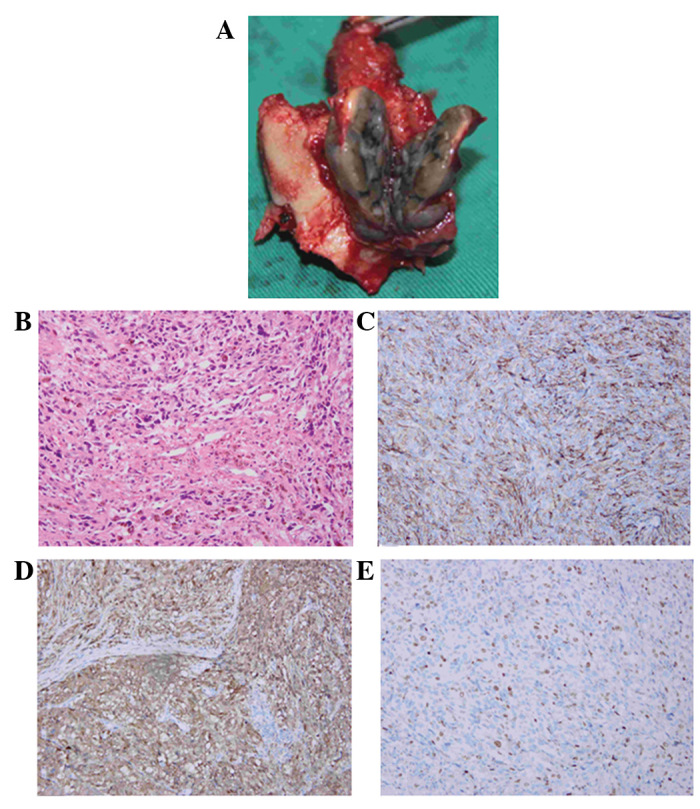
(A) The tumor was dark gray and located in front of the L3 lamina. (B) Certain tumor cells were pigmented (HE staining; magnification, 10×20). (C) Diffuse distribution of anti-HMB-45 reactive tumor cells. (PAP staining; magnification, 10×20). (D) Tumor cells stained markedly positive for S-100 protein. (PAP staining; magnification, 10×20) (E) Positive Ki-67 index detected in ∼25% of cells. (PAP staining; magnification, 10×20). HE, hematoxylin and eosin; PAP, peroxidase-antiperoxidase.

**Figure 3:**
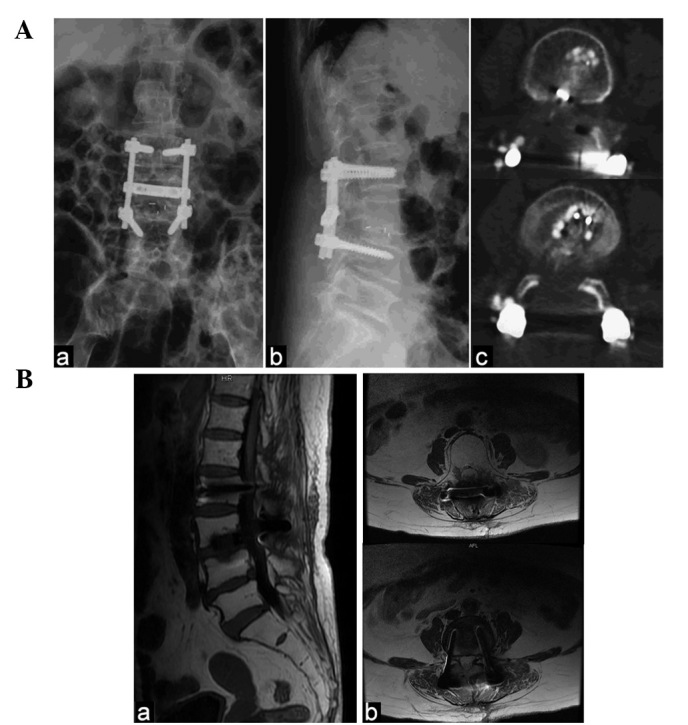
(A) Post-surgery (a and b) lumbar radiograph and CT scan. (c) Removal of the tumor, L3 lamina and inferior articular process and spine fixation at the L2–4 level and interbody fusion at the L3–4 level. (B) At 13 months post-surgery, (a and b) the MRI showed no evidence of recurrence. Aa, posteroanterior; Ab, lateral; Ac, transverse plane; Ba, T1/sagittal plane; Bb, T1/transverse plane. CT, computed tomography; MRI, magnetic resonance imaging.

A posterior laminectomy and fusion with instrumentation were attempted on the patient. However, during the surgery, a dark gray solid mass was observed under the L3 vertebral plate. The mass was dissected carefully from the surrounding structures and gross total removal with the L3 lamina and inferior articular process was performed. The solid mass (5.0×4.7×3.0 cm) was located between the L3 lamina and dura and was ellipsoid in shape. The mass appeared to be wrapped in a membrane and adhered to the dorsal dural surface (Fig. 2A). Spine fixation was subsequently performed at the L2-4 level and interbody fusion was performed at the L3-4 level (Fig. 3A).

The hematoxylin and eosin (HE) histological analysis revealed a pigmented tumor (Fig. 2B), while the immunohistochemical analysis revealed cells stained markedly positive for the monoclonal antibody anti-HBM-45, which is a useful marker of melanocytic differentiation in neoplasms (Fig. 2C). The S-100 protein stain, a marker for cells derived from the neural crest, was positive in the neoplastic cells (Fig. 2D). Furthermore, the Ki-67 index was positive in ∼25% of the cells. (Fig. 2E). The pathological diagnosis based on these observations was one of malignant melanoma.

Subsequent to the surgery, the general condition of the patient was good with no lower back pain. At two weeks post-surgery, bone scans showed an increased radioactive uptake, not only at L3, but also at the right sacroiliac joint. The patient was subsequently treated with radiotherapy and immunotherapy. The metastatic melanoma was absent in all areas, including the right sacroiliac joint, following this comprehensive therapy. At the final 13-month follow-up, the patient showed no evidence of recurrence and the intermittent claudication had disappeared (Fig. 3B).

## Discussion

Melanomas occurs most commonly in Caucasian populations and are rare in Asian populations (4). However, the incidence of melanomas is also rising in each population (5,6). Melanomas are prone to early distant metastasis involving almost any organ or structure, and following the development of a distant metastasis, the prognosis is poor (1). The most common forms of metastatic melanoma of the spine are vertebral metastatic melanoma and intramedullary metastatic melanoma. Gokaslan *et al*(2) reported 133 cases of vertebral metastatic melanoma over a period of 11 years and Ishii *et al*(3) reviewed reports of nine cases of intramedullary spinal cord metastatic melanoma. In the present case, the metastatic melanoma was located in the extradural space of the spinal canal and the metastasis was detected 13 years after the vulvar melanoma resection. For these reasons, a diagnosis of metastatic melanoma was not initially considered.

MRI is an optional method for the diagnosis of spinal tumors that aids in the diagnosis of spinal melanoma. In a case of vertebral metastatic melanoma, MRI reveals an increased signal intensity in the vertebral body, with soft-tissue extension into the extradural space (2,7). MRI scans of intraspinal melanomas show hypointense signals on T2-weighted images and hyperintense signals on T1-weighted images. Unal and Castillo (8) reported a primary thoracic extradural spinal malignant melanoma with T2-hypointense and T1-hyperintense MRI signals. Lee *et al*(9) presented the MRI analysis of a patient with a primary intradural extramedullary melanoma of the cervical spinal cord, with decreased signal intensity on T2-weighted images and increased signal intensity on T1-weighted images. Lee *et al*(10) reported a case in which the MRI of the patient revealed an enhanced mass in the intra- and extradural space compressing the spinal cord at the left neural foramen at the C6-7 level. However, MRI does not consistently show a homogeneous pattern. The MRI signal of melanocytic tumors depends on the presence of melanin, acute or chronic intratumoral hemorrhages and fat deposits. Therefore, as in the present case, the interpretation of the MRI pattern may easily lead to misdiagnosis (11). MRI of the lumbar spine of the present patient showed a mass with low intensity on T2-weighted images, mixed low signal intensity on T1-weighted images and slight enhancement following gadolinium-contrast injection. Consequently, lumbar stenosis resulting from hypertrophy of the ligamentum flavum was initially suspected as it is a common disease in older individuals.

Usually, the confirmatory diagnosis of spinal melanoma is only made on the basis of post-surgical pathological studies or autopsies (3,7–9,12,13). Immunohistochemical studies are also important in a diagnosis. Anti-melanoma antibody (HMB-45) and S-100 protein staining may aid in the diagnosis of malignant melanoma. Furthermore, immunohistochemistry may be used to distinguish spinal melanoma from other types of tumors (9,14). In the present case, staining for the translocation factor E3, smooth muscle actin, pan-cytokeratin, desmin and collagen IV was negative, thus aiding in the differential diagnosis.

Surgical resection of spinal melanomas is extremely important as it leads to the regression of neurological symptoms (13). Although the efficacies of radiotherapy and chemotherapy remain controversial in melanoma, radical removal of the tumor should be followed by radiotherapy due to the malignant nature of this tumor (9,15,16). The present patient received post-surgery radiotherapy and immunotherapy, not only for the treatment of the extradural melanoma of the lumbar spine, but also for the melanoma of the right sacroiliac joint. With comprehensive treatment, patient survival-times range between one week and 43 months following the diagnosis of vertebral metastatic melanoma, and between eight weeks and 18 years following the initial presentation in patients with intramedullary metastatic melanoma (2,7,8,17,18). Although the present patient remains alive and in a good condition at the 13th-month follow-up, we propose that an earlier surgery instead of the treatment with medication would have been beneficial for the patient’s survival and health.

In summary, due to the delayed metastasis, location and unusual appearance of the tumor in the MRI scans, a metastatic melanoma was not initially suspected in the present case. Early surgical removal of the tumor allows tissue diagnosis and the selection of an appropriate comprehensive treatment, which is critical for patient survival. The present case indicates that caution should be used in the diagnosis of similar future cases.

## References

[1] O’Day SJ, Kim CJ, Reintgen DS (2002). Metastatic melanoma: chemotherapy to biochemotherapy. Cancer Control.

[2] Gokaslan ZL, Aladag MA, Ellerhorst JA (2000). Melanoma metastatic to the spine: a review of 133 cases. Melanoma Res.

[3] Ishii T, Terao T, Komine K, Abe T (2010). Intramedullary spinal cord metastases of malignant melanoma: an autopsy case report and review of the literature. Clin Neuropathol.

[4] Crombie IK (1979). Racial differences in melanoma incidence. Br J Cancer.

[5] Jemal A, Siegel R, Ward E (2008). Cancer statistics, 2008. CA Cancer J Clin.

[6] Ishihara K, Saida T, Otsuka F, Yamazaki N, Prognosis and Statistical Investigation Committee of the Japanese Skin Cancer Society (2008). Statistical profiles of malignant melanoma and other skin cancers in Japan: 2007 update. Int J Clin Oncol.

[7] Eck JC, Tressler MA, Triantafyllou SJ (2008). Delayed presentation of metastatic melanoma of the cervical spine. Orthopedics.

[8] Unal B, Castillo M (2007). MRI features of a primary thoracic epidural melanoma: a case report. Clin Imaging.

[9] Lee CH, Moon KY, Chung CK, Kim HJ, Chang KH, Park SH, Jahng TA (2010). Primary intradural extramedullary melanoma of the cervical spinal cord: case report. Spine (Phila Pa 1976).

[10] Lee NK, Lee BH, Hwang YJ (2010). Findings from CT, MRI, and PET/CT of a primary malignant melanoma arising in a spinal nerve root. Eur Spine J.

[11] Farrokh D, Fransen P, Faverly D (2001). MR findings of a primary intramedullary malignant melanoma: case report and literature review. AJNR Am J Neuroradiol.

[12] Vij M, Jaiswal S, Jaiswal AK, Behari S (2010). Primary spinal melanoma of the cervical leptomeninges: report of a case with brief review of literature. Neurol India.

[13] Kolasa M, Jesionek-Kupnicka D, Kordek R, Kolasa P (2010). Primary spinal cord melanoma - a case report. Folia Neuropathol.

[14] Kounin GK, Romansky KV, Traykov LD, Shotekov PM, Stoilova DZ (2005). Primary spinal melanoma with bilateral papilledema. Clin Neurol Neurosurg.

[15] François P, Lioret E, Jan M (1998). Primary spinal melanoma: case report. Br J Neurosurg.

[16] Narayan RK, Rosner MJ, Povlishock JT, Girevendulis A, Becker DP (1981). Primary dural melanoma: a clinical and morphological study. Neurosurgery.

[17] Connolly ES, Winfree CJ, McCormick PC, Cruz M, Stein BM (1996). Intramedullary spinal cord metastasis: report of three cases and review of the literature. Surg Neurol.

[18] Nishihara M, Sasayama T, Kondoh T, Tanaka K, Kohmura E, Kudo H (2009). Long-term survival after surgical resection of primary spinal malignant melanoma. Neurol Med Chir (Tokyo).

